# Ultrasound-Guided Cervical Selective Nerve Root Block versus Fluoroscopy-Guided Interlaminar Epidural Injection for Cervical Radicular Pain: A Randomized, Prospective, Controlled Study

**DOI:** 10.3390/jpm14070721

**Published:** 2024-07-04

**Authors:** Halil Cihan Kose, Selin Guven Kose, Feyza Celikel, Serkan Tulgar, Omer Taylan Akkaya

**Affiliations:** 1 Department of Pain Medicine, Health Science University Kocaeli City Hospital, 41060 Kocaeli, Turkey; selinguven89@gmail.com; 2 Department of Physical Therapy and Rehabilitation, Sakarya Training and Research Hospital, 54120 Sakarya, Turkey; 3 Department of Anesthesiology and Intensive Care, Samsun University Samsun Training and Research Hospital, 41060 Samsun, Turkey; serkantulgar.md@gmail.com; 4 Department of Pain Medicine, Ankara Etlik City Hospital, 06220 Ankara, Turkey; taylanakkaya.md@gmail.com

**Keywords:** selective nerve root block, chronic pain, radicular pain, ultrasound, cervical radiculopathy, steroid, numeric rating scale

## Abstract

Ultrasound (US)-guided cervical selective nerve root block (CSNRB) procedures are increasingly being performed as an alternative to conventional fluoroscopy (FL)-guided epidural injections for the treatment of cervical radicular pain. The aim of this study was to compare the effectiveness of US-guided CSNRB versus FL-guided interlaminar cervical epidural steroid injection (IL-CESI) for cervical radicular pain. A total of 60 patients with cervical radicular pain due to a single-level disc herniation were randomized into either the FL or US group. The numeric rating scale, Short Form-36, and neck disability index were evaluated before treatment at months 1, 3, and 6 after treatment. Procedure time, complications, pain medication consumption, and patient satisfaction were also recorded. Patients experienced significant improvement in pain, disability, and quality of life scores up to 6 months after the procedure (*p* < 0.001). Treatment success rate was achieved in 56.6% of the IL-CESI group and 50% of the CSNRB group without any significant difference between the study arms (*p* = 0.617). US-guided CSNRB was shown to be as effective as the FL-guided IL-CESI in the treatment of cervical radicular pain, in addition to the absence of radiation exposure and requiring less procedure time.

## 1. Introduction

Cervical radicular pain is a major problem worldwide, with an estimated annual incidence rate of 107.3 for males and 63.5 for females per 100,000 population, and it exerts an enormous personal and socioeconomic burden [1]. One of the most common causes of radicular pain in the upper extremities is cervical disc herniation (CDH), the symptoms of which usually affect the quality of life, functional capacity, and sleep [2]. The pathophysiology of cervical radicular pain is considered to be a combination of mechanical compression and chemical irritation of the nerve roots [3]. In general, epidural injections through interlaminar and transforaminal routes are recommended if medical therapy, physical therapy, and lifestyle-modifying treatments have failed [4,5]. To date, several trials have been performed to compare the effectiveness and safety of these two approaches, but debate remains as to which approach is superior to the other [6,7,8,9,10]. Many physicians assert that fluoroscopy (FL)-guided transforaminal epidural steroid injection (TF-ESI) has the advantage of accurately delivering the injectate into the anterior epidural space, optimizing the concentration of medication at the site of pathology. On the basis of the current literature, there are reports of potentially serious complications such as spinal cord injury or vertebral artery dissection, even with strict guidelines [11,12]. Additionally, severe complications such as epidural hematoma and spinal cord injury have raised concerns and questioned the prevailing belief that interlaminar cervical epidural steroid injection (IL-CESI) is safer than TF-ESI [13,14]. As a result, controversy remains over the most optimal technique for cervical radicular pain due to safety concerns.

Recently, there has been a shift from FL to ultrasound (US) to guide interventional procedures [15,16,17]. The major advantages of US guidance include real-time visualization and avoidance of radiation exposure. US-guided cervical selective nerve root block (CSNRB) is both a diagnostic and therapeutic approach used for cervical radicular pain. In CSNRB, the specific target is the affected nerve root, particularly the ventral ramus, located in the transverse process groove between the anterior and posterior tubercles. Unlike the FL-guided TF-ESI technique, in CSNRB, the needle is advanced to the extraforaminal area in order to avoid vascular structures within the foramen and is not intended to administer the medication into the epidural space [18]. Recently, several research has shown the reliability and effectiveness of US-guided CSNRB [19,20,21,22,23].

The primary objective of this prospective, randomized-controlled trial was to compare the effects of US-guided CSNRB versus FL-guided IL-CESI in patients with cervical radicular pain that persisted for at least three months due to single-level CDH on pain at six months after treatment. Patients with a history of previous cervical spine surgery, multi-level herniated disk, or multi-level cervical radicular pain were excluded from the study. Functional disability, quality of life, patient satisfaction, analgesic use, and procedure time comprised secondary outcomes.

## 2. Materials and Methods

### 2.1. Design

In accordance with the Helsinki Declaration, this trial was conducted as a prospective, randomized, assessor-blinded trial, and approval was obtained from the ethics committee of Diskapi Yildirim Beyazit Training and Research Hospital (131/10-21.02.2022). All subjects provided written informed consent. The trial was registered on ClinicalTrials.gov in April 2022 (NCT05340179). This trial was conducted between April 2022 and April 2023. 

### 2.2. Patients

Inclusion criteria were cervical radicular pain that persisted for at least 3 months, ≥18 years, unilateral single-level cervical radicular pain based on medical examination and confirmation of a single-level CDH via magnetic resonance imaging, numeric rating scale (NRS) score of ≥4, refractory pain after conservative treatment. Exclusion criteria were segmental instability/scoliosis/spondylolisthesis, body mass index ≥ 30 kg/m^2^, neuropsychiatric disease, epidural steroid injection within the past 6 months, previous cervical spine surgery, multi-level herniated disk, multi-level cervical radicular pain, neurological deficit, allergic reactions to contrast medium, pregnancy or contraindications to epidural injection. Patients were advised to continue taking their analgesics, if necessary, throughout the follow-up period.

### 2.3. Randomization and Blinding

A computer-generated randomization schedule was utilized for randomization (https://www.randomizer.org/, accessed on 30 April 2022). Patients were assigned a number and then allocated into two groups, with 30 patients in each group. Throughout the trial, the evaluator remained unaware of the randomization process, ensuring blinding.

### 2.4. Interventions

#### 2.4.1. IL-CESI Group

The patient was positioned in the prone position with a pillow under the chest to raise the shoulders and flex the spine. After sterile cleaning and covering, 0.5–1 mL of 1% lidocaine was administered subcutaneously. Using a C-arm, an 18G tuohy needle was used to access the epidural space between C6, C7, and T1 via a paramedian approach. Once the needle was positioned just posterior to the spinolaminar line in lateral view, it was carefully advanced, and the ligamentum flavum was punctured using a loss-of-resistance technique. Confirmation of correct needle tip placement in the epidural space was confirmed through anteroposterior and lateral FL views, with contrast material flowing through the epidural space. Subsequently, a 3 mL drug was injected into the epidural space, comprising 2 mL of dexamethasone (8 mg) and 1 mL of 2% lidocaine.

#### 2.4.2. CSNRB Group

**Figure 1:**
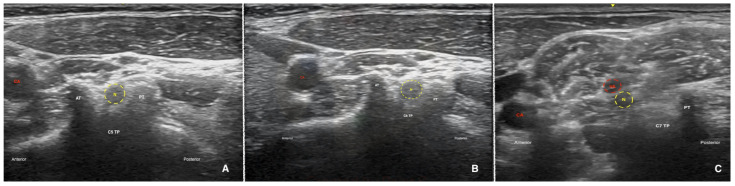
Ultrasound images of the C5 (**A**), C6 (**B**) and C7 (**C**) nerve roots. (**A**) Sonographic image showing the anterior tubercle (AT) and the posterior tubercle (PT) of the C5 cervical level (two-humped camel signs). (**B**) Sonographic image showing the taller AT (Chassaignac tubercle) and the PT of the C6 cervical level. (**C**) Sonographic image showing the C7 cervical level, which has only a PT. N—nerve root, CA—carotid artery, TP—transverse process, VA—vertebral artery.

The procedure was performed in the lateral decubitus position. A high-resolution (2–12 Hz) linear US probe was positioned transverse to the side of the neck, and the cervical tubercles were visualized. The nerve roots were identified by the anatomy of the transverse process; the C5th level has the same heights of anterior and posterior tubercles (Figure 1A); C6th level, the anterior tubercle is relatively taller, while the posterior tubercle is significantly shorter (Figure 1B); C7th has a prominent posterior tubercle and a rudimentary anterior tubercle (Figure 1C).

After the cervical spine levels were determined, the US probe was tilted to clarify the target nerve root. The hypoechoic cervical spinal nerve can be identified in this view between the hyperechoic posterior and anterior tubercle. After performing subcutaneous local anesthetic, the needle was inserted from posterior to anterior using an in-plane approach to target the nerve root to the extraforaminal space. The target was directly posterior to the nerve root. A color doppler was performed to avoid penetration of vessels. After negative aspiration, 1 mL of 1% lidocaine as a test dose was administered to prevent intravascular injection, and the patient was monitored for 2–3 min. After verifying the absence of abnormal findings, 3 mL of solution (1 mL of 2% lidocaine, 2 mL of dexamethasone 4 mg/mL) was administered.

### 2.5. Outcome Measurements

Baseline data, including age, sex, and body mass index, were collected. Follow-up assessments were performed at months 1, 3, and 6 after the procedure. For evaluation of pain severity, patients were asked to report their average pain over the past week on the NRS [24]. As a primary outcome measure, the NRS pain score was evaluated six months after treatment. Secondary outcomes included the percentage of successful responders (50% or greater improvement in NRS) and subjects who achieved a minimal clinically important difference (MCID) in NRS, functional status, quality of life, analgesic use, and patient satisfaction. The MCID for the NRS scores was determined by using threshold scores established in the previous studies: an improvement in NRS of at least 2 points [25].

Functional status was assessed with the neck disability index (NDI) [26]. The Turkish version of the SF-36 was utilized to evaluate quality of life. The SF-36 provides the physical component summary (PCS) and the mental component summary (MCS) [27]. To assess pain medication regimens and track changes, the quantitative analgesic questionnaire (QAQ) was performed [28]. Overall satisfaction was assessed using a 5-point Likert scale ranging from 1 (very dissatisfied) to 5 (very satisfied) [29]. Procedure time and side effects were also recorded. For CSNRB, this was the time from the moment the US probe touched the patient’s skin until the injection was completed. For IL-CESI, this was the time from obtaining the first radiographic image to completing the injection.

The degree of compression of the nerve root established by Klessinger et al. was evaluated using T2-weighted axial MRI scans [30]. According to this classification, grade I defines no contact between the disc and nerve root, and grade II defines disc contact to the nerve without displacement or compression. Grade III was assigned if the nerve root was displaced but not compressed, and grade IV indicates the compressed and morphologically distorted nerve root.

### 2.6. Sample Size Determination

The sample size calculation was performed using G *Power software version 3.1.9.7 (Heinrich-Heine-Universität, Düsseldorf, Germany) based on the findings of a previous study. The mean NRS score was 3.9 ± 1.5 at 6 months after IL-CESI in that study [31]. With the NRS as the primary outcome, a sample size of 25 patients was calculated to be required to detect a 30% difference, with a significance level of 0.05 and a power of 80%. Taking into account the potential loss of 20% of patients during the follow-up period, a sample size of 30 patients was established for each group.

### 2.7. Statistical Analysis

Statistical analysis was performed with IBM SPSS Statistics version 20. Continuous variables were reported as mean with standard deviation or median with interquartile range, while categorical variables were reported as counts and percentages. The normal distribution of the data was assessed using the Shapiro–Wilk test. The χ^2^ test or Fisher exact test was used to compare categorical variables. For non-normally distributed data, the Mann–Whitney U test was used for comparison, while the independent t-test was used for normally distributed data. Two-way repeated measures analysis of variance was used to determine changes from baseline for variables at each time point within and between groups, with post-hoc Bonferroni tests. A *p*-value of <0.05 was considered statistically significant.

## 3. Results

In this study, 60 patients were randomly allocated to the treatment groups. No significant differences were observed between the groups in baseline demographic data or severity of nerve root compression (*p* > 0.05) (Table 1). 

The group allocation did not have a significant effect on the NRS scores within groups [F (1, 58) = 0.687, *p* = 0.411], as well as on the NDI scores [F (1, 58) = 0.838, *p* = 0.364] (Table 2). A significant effect of time was found in the NRS [F (2.57, 149.27) = 211.257, *p* < 0.001] and NDI [F(2.033, 117.938) = 103,097, *p* < 0.001] in both groups. No significant interaction was presented between time and group allocation for NRS [F(2.57, 149.27) = 0.658, *p* = 0.556] and NDI [F(2.033, 117.938) = 0.251, *p* = 0.782] scores (Table 2 and Table 3). 

Both treatment groups showed significant improvement in NRS, NDI, and SF-36 scores at each follow-up point during the six months compared with pre-intervention (*p* < 0.001) (Table 3). Treatment success, defined as the percentage of patients experiencing more than 50% pain reduction, was achieved in 56.6% (*n* = 17) of the IL-CESI group and 50% (*n* = 15) of the CSNRB group at the end of the study. At month 6, it was observed that 86.7% (26 out of 30) of patients in the IL-CESI group experienced an MCID in NRS compared with 83.3% (25 out of 30) in the CSNRB group. However, no significant difference was found between the groups (Table 2).

In both groups, a significant decrease in analgesic use was observed at six months in comparison with baseline (*p* < 0.001). Among the patients who underwent IL-CESI and CSNRB, 80% and 73.3% reported being very satisfied or satisfied, respectively. However, no significant differences were found between the groups (*p* > 0.05). Procedure time was significantly shorter in the US-guided CSNRB (352.42 ± 103.61) than in the FL-guided IL-CESI (208.76 ± 96.2) (*p* < 0.001) (Table 3).

The manifestation of blood aspiration before injection was observed in three patients in the FL-guided group but in none in the US-guided group. Intravascular contrast spread during injection was observed in two patients of the FL-guided group. For US-guided CSNRB, an additional contrast agent was not performed to detect intravascular spread with FL. Adverse events immediately after the procedure, such as dizziness, nausea, vomiting, and vasovagal reactions, were observed in three patients (10%) and four patients (13.3%) in the US group and FL group, respectively (*p* = 0.687). No serious complications, such as infection, hematoma, or motor deficit, were observed. 

## 4. Discussion

The present study investigated the efficacy of US-guided CSNRB versus FL-guided IL-CESI in unilateral cervical radicular pain due to a single-level CDH. The results of this study suggest that US-guided CSNRB is a feasible alternative treatment option. Patients in both groups showed similar treatment outcomes in terms of pain relief, functional disability, quality of life, and pain medication consumption over six months. The proportion of subjects who experienced an MCID and a positive treatment outcome on the NRS was not statistically different between the groups. However, procedure time was higher in the IL-CESI group.

ESI through interlaminar and transforaminal routes under FL guidance is an effective approach to close the gap between conservative treatment modalities and cervical spine surgery in cervical radicular pain, but a debate is still ongoing about the superiority of one over the other. Generally, physicians avoid performing IL-CESI over IL-lumbar ESI because the cervical epidural area anatomy is somewhat different. The cervical epidural space has the smallest size among all spinal levels [32]. Additionally, epidural fat is unevenly distributed and almost non-existent at the cervical level [33]. TF-ESI is a more targeted-selective treatment option for cervical radicular pain. However, the anatomical proximity between the vertebral arteries and the cervical neuroforamen plays a significant role in injury to the vertebral arteries or the occurrence of neurologic complications [34]. Additionally, the TF-ESI technique does not reduce the risk of potentially serious complications attributed to IL-CESI, such as hematoma and subdural or dural punctures [35]. The role cervical ESI plays across a spectrum of resolution of radicular symptoms is clear and well documented. However, challenges remain regarding the translation of these substantial benefits into a safer treatment approach. 

Recently, there has been a shift from FL to US for guiding spinal procedures, as the latter can provide direct-time visualization and can help confirm the location of nerves, vessels, and injections. This advantage is particularly valuable for cervical interventions, as this area contains numerous vulnerable vessels and vital structures, and the path of the needle during injection often crosses these structures. Considering these safety concerns, US imaging is a safe and reliable technique for CSNRB to provide well-defined images of the cervical neuroforamen with continuous visualization, which could decrease the reported complications. In US-guided CSNRB, the needle is placed in the intertubercular neural groove between the anterior and posterior tubercles of the cervical vertebra outside the foramen of the affected nerve without entering the epidural space. After the initial description of the US-guided CSNRB, priorities for research include treatment outcomes and complications in cervical radicular pain.

In the present study, no difference in treatment outcomes was found regarding improvement in pain relief, quality of life, and functional capacity during the follow-up period when the US-guided CSNRB and FL-guided IL-CESI were compared. In a prospective randomized study, Jee et al. investigated the efficacy of US-guided CSNRB versus FL-guided TF-ESI in cervical radicular pain. They reported that the US-guided CSNRB provided similar improvement in pain scores and functional disability during the 3-month follow-up period as the FL-guided approach [21]. In another retrospective study, Jang et al. evaluated the effectiveness of FL-guided CESI and TF-ESI and US-guided CSNRB. The findings of their trial showed that the outcomes of the three procedures were similar in terms of pain relief, improvement in functionality, and reduction in the percentage of analgesic users during the 6-month follow-up [35]. In a retrospective comparative study, Park et al. showed that US-guided CSNRB provides a similar improvement in pain scores and functional disability as FL-guided IL-CESI [36]. To the best of our knowledge, no previous prospective randomized controlled studies have investigated the effects of US-guided CSNRB on pain medication consumption, patient satisfaction, and functional improvement. Additionally, there have been no comparisons using the aforementioned parameters between US-guided CSNRB and FL-guided CESI. Based on the literature, including this study, US-guided CSNRB can be an alternative treatment modality in cervical radicular pain due to its similar effects to FL-guided procedures.

Although there is no standardization, injection volumes between 1 and 4 mL are recommended for CSNRB. In the context of CSNRB, while it is presumed that spread is limited to the targeted nerve root and does not result in an unexpected epidural block at other spinal levels, high volume, low viscosity, technical factors, hydrostatic pressure, and osmotic effects, an extension of dural sleeve into the foramen may result in neuraxial spread. Recently, Ma et al. investigated injection spread patterns of US-guided CSNRB with a 3 mL injection. They reported that contrast spread into the extraforaminal area in 100% of patients and to the intraforaminal area in 61.90% of patients on post-procedure CT scan images, and finally to the epidural spaces in only 9.52%, with contrast spread limited to the corresponding cervical level [23]. In another study, Kang et al. investigated the spread pattern related to injection volume. The study revealed that none of the patients who had received a 1 mL injection of contrast medium exhibited diffusion into the epidural area, whereas 13 subjects (24.5%) who had received a 4 mL injection showed diffusion into the intraforaminal epidural area [37]. However, there was no relationship between the improvements in pain scores and the spread pattern of contrast. In this study, a 3 mL treatment drug was chosen according to our routine clinical practice. Similar to our results, two retrospective studies demonstrated that US-guided CSNRB performed with a 3 mL injection consisting of dexamethasone and lidocaine is effective for cervical radicular pain for up to 6 months [35,36]. 

The use of ultrasound is beneficial in identifying atypical vessels that may be located unexpectedly proximal to the intervertebral foramen. However, it may not be able to detect intravascular injections. In this present study, color Doppler US, a non-particulate steroid, and a test dose by administering one mL of 1% lidocaine prior to US-guided CSNRB were performed to prevent potential complications associated with intravascular injection. During the FL-guided CESI procedure, intravascular injection and blood aspiration before injection were identified in two and three patients, respectively. However, blood aspiration before injection was not noted during the US approach. Although we did not use an additional contrast agent with FL to confirm the appropriate injection of the medication during US-guided CSNRB, we found no intravascular events associated with the procedure, which is in line with the findings of previous studies [21,35,36,38]. It is important to note that the US may not always reliably detect microvascular injections, which can potentially result in neurological complications. Therefore, it is still necessary to exercise caution when using current US technology to confirm the absence of small critical vessels. As a result, we suggest performing a lidocaine test, color Doppler, and non-particulate steroids to support the safety of the US approach.

The findings of this study favored US-guided CSNRB over FL-guided IL-CESI in terms of procedure time, which is consistent with previous reports [35,36]. Many physicians may encounter difficulties in accessing FL devices and shielding leads in operating rooms. US assistance not only allows radiation-free imaging and shorter procedure times but also offers advantages in terms of greater portability and affordability over FL. On the other hand, it is important to note that this study included subjects with unilateral single-level cervical radicular pain due to single-level CDH. When IL-CESI is performed, the injection results in the blockade of nerve roots at other levels. Therefore, in patients with bilateral cervical radicular pain or patients with pain in more than one dermatomal distribution, safety concerns and procedure time must be taken into account when performing a US-guided CSNRB.

It is important to acknowledge some limitations of this study. First, the trial was not designed as a double-blinded study; it is challenging to conduct a double-blinded study with imaging devices such as US or FL. Further research comparing these imaging techniques could ensure blinding by scanning patients with a mock US. Second, different NRS scores could be used for neck and arm pain. Third, the sample size is relatively small, and the study’s follow-up period was of short duration, specifically limited to a six-month post-treatment evaluation period. Fourth, the results could have been strengthened if contrast spread to the affected nerve root during US-guided CSNRB had been verified. Fifth, the treatment procedures were performed by a single physician, and the physician’s experience in obtaining good images and safely directing the needle to the target point may have influenced our results. Sixth, it was hypothesized that pain relief might last longer in patients following ESI of particulate steroids compared to non-particulate steroids. We used dexamethasone based on previous studies and discussion among the authors of this study to provide similarity between groups, although there is no evidence to suggest that using non-particulate dexamethasone offers any safety advantage over particulate steroids [39]. Seventh, the natural course of cervical radiculopathy is difficult to outline. Due to the absence of a placebo/sham group, we can not exclude the natural course of the disease or the placebo effect of the treatments. It is worth noting that the prevalence of placebo and nocebo effects in the context of interventional treatments is estimated to range between 13% and 30% and 3% and 8%, respectively [40]. Within the scope of this study, it is important to emphasize that all enrolled patients had not responded to conventional therapeutic modalities. Considering these observations and the inherently progressive and degenerative nature of refractory chronic radicular pain, we assert that the pain experienced by our patients has reached a plateau. Finally, the US approach was performed in patients with a BMI less than 30 kg/m^2^. Visualization of small vessels such as radicular arteries may be very difficult, especially in obese patients.

## 5. Conclusions

The US-guided CSNRB was shown to be as effective as the FL-guided IL-CESI in the treatment of cervical radicular pain, in addition to the absence of radiation exposure and requiring less procedure time. Future studies are needed to investigate the potential of US-guided CSNRB and confirm or refute the findings of this study.

## Figures and Tables

**Table 1:** General characteristics of the patients.

Variables	IL-CESI (*n* = 30)	CSNRB (*n* = 30)	*p* Value
Age (years)	54.23 ± 8.98	55.1 ± 10.03	0.555
Sex			
Male	14 (46.7%)	17 (56.7%)	0.605
Female	16 (53.3%)	13 (43.3%)	
BMI (kg/m^2^)	24.02. ± 2.47	23.13 ± 2.52	0.914
Affected side			
Left	17 (56.7%)	15 (50%)	0.796
Right	13 (43.3%)	15 (50%)	
Pain duration (months)	11.5 ± 5.83	10.13 ± 4.25	0.094
Target nerve root			
C5	3 (10%)	2 (6.7%)	0.716
C6	17 (56.7%)	15 (50%)	
C7	10 (33.3%)	13 (43.3%)	
Grade of nerve root compression			
Grade II	19 (63.3%)	15 (50%)	0.550
Grade III	10 (33.3%)	13 (43.3%)	
Grade IV	1 (3.3%)	2 (6.6%)	

Data are in mean ± standard deviation, ratio, number (percentage), and median (interquartile range). BMI—body mass index, IL-CESI—interlaminar cervical epidural steroid injection, CSNRB—cervical selective nerve root block.

**Table 2:** Comparison of the pain scores.

	IL-CESI	CSNRB	*p*
NRS			
Baseline	7 (6–8)	6.5 (6–7.75)	0.105
Month 1	1.5 (1–2) *	1.5 (1–2.75) *	0.776
Month 3	2 (2–3) *	2 (2–2.75) *	0.789
Month 6	3.5 (2–4) *	3 (2–4) *	0.927
Positive responders			
Month 1			
*n* (%) (95% CI)	25 (83.3%) (65.2–94.3)	24 (80%) (61.4–92.2)	0.753
Month 3			
*n* (%) (95% CI)	22 (73.3%) (54.1–87.7)	23 (76.6%) (57.7–90)	0.776
Month 6			
*n* (%) (95% CI)	17 (56.6%) (37.4–74.5)	15 (50%) (31.3–68.7)	0.617
NRS MCID			
Month 1			
*n* (%) (95% CI)	29 (96.7%) (82.7–99.9)	28 (93.3%) (77.9–99.1)	0.618
Month 3			
*n* (%) (95% CI)	27 (90%) (73.4–97.8)	26 (86.7%) (69.2–96.2)	0.711
Month 6			
*n* (%) (95% CI)	26 (86.7%) (69.2–96.2)	25 (83.3%) (65.2–94.3)	0.735

Data are in median (25th–75th percentiles) or numbers (percentages) (95% CI). CSNRB—cervical selective nerve root block, IL-CESI—interlaminar cervical epidural steroid injection, MCID—minimally clinically important difference, NRS—numeric rating scale. A positive response was defined as ≥50% reduction from baseline NRS score. *p*-values compare scores between the groups. (*) statistically significant change in comparison to the baseline value within the same group; *p* < 0.001. MCID indicates the proportions of each study group, defined as the percentage of each study arm that had an MCID decrease in the NRS score (2 points).

**Table 3:** Follow-up of outcomes in the treatment groups.

Variables	IL-CESI (*n* = 30)	CSNRB (*n* = 30)	*p* Value
NDI			
Baseline	30.3 ± 12.11	28.23 ± 10.29	0.479
Month 1	13.7 ± 5.39 *	13.63 ± 5.72 *	0.963
Month 3	12.2 ± 7.45 *	10.93 ± 5.26 *	0.450
Month 6	12.9 ± 6.05 *	11.5 ± 4.52 *	0.315
PCS			
Baseline	32.15 ± 20.23	29.84 ± 18.75	0.648
Month 1	39.34 ± 9.36 *	37.25 ± 10.23 *	0.412
Month 3	49.55 ± 8.96 *	50.12 ± 9.72 *	0.814
Month 6	47.83 ± 9.21 *	46.93 ± 10.54 *	0.726
MCS			
Baseline	49.05 ± 17.93	47.26 ± 16.95	0.692
Month 1	52.62 ± 10.91 *	49.33 ± 12.25 *	0.276
Month 3	58.76 ± 11.34 *	54.35 ± 13.21 *	0.170
Month 6	56.42 ± 11.21 *	52.97 ± 12.34 *	0.261
QAQ			
Baseline	4.93 ± 2.03	4.46 ± 1.87	0.661
Month 6	1.63 ± 0.66 *	1.46 ± 0.68 *	0.329
Patient Satisfaction			
Month 6	4 (4–5)	4 (3.25–5)	0.374
Procedure Time			
Seconds	208.76 ± 96.2	352.42 ± 103.61	<0.001

Values are presented as means ± standard deviations, median (percentiles 25–75). IL-CESI—interlaminar cervical epidural steroid injection, CSNRB—cervical selective nerve root block, NDI—neck disability index, MCS—mental component score, PCS—physical component score, QAQ—quantitative analgesic questionnaire. (*) *p* < 0.05 compared with baseline values in each group. *p*-values comparing scores between both groups.

## Data Availability

Present with the corresponding author and can be delivered upon reasonable request.
